# Food Choice Determinants and Perceptions of a Healthy Diet among Italian Consumers

**DOI:** 10.3390/foods10020318

**Published:** 2021-02-03

**Authors:** Rungsaran Wongprawmas, Cristina Mora, Nicoletta Pellegrini, Raquel P. F. Guiné, Eleonora Carini, Giovanni Sogari, Elena Vittadini

**Affiliations:** 1 Department of Food and Drug, University of Parma, Parco Area delle Scienze 47/A, 43124 Parma, Italy; rungsaran.wongprawmas@unipr.it (R.W.); eleonora.carini@unipr.it (E.C.); giovanni.sogari@unipr.it (G.S.); 2 Department of Agricultural, Food, Environmental and Animal Sciences, University of Udine, via Sondrio 2/A, 33100 Udine, Italy; Nicoletta.pellegrini@uniud.it; 3 CERNAS Research Centre, Polytechnic Institute of Viseu, 3504-510 Viseu, Portugal; raquelguine@esav.ipv.pt; 4 School of Biosciences and Veterinary Medicine, University of Camerino, Via Gentile III da Varano, 62032 Camerino, Italy; elenagiovanna.vittadini@unicam.it

**Keywords:** food choices, eating determinants, healthy diet, emotions

## Abstract

Healthy food choices are crucial for a healthy lifestyle. However, food choices are complex and affected by various factors. Understanding the determinant factors affecting food choices could aid policy-makers in designing better strategies to promote healthy food choices in the general public. This study aims to evaluate the food choice motivations and to segment consumer groups, according to their food choice motivations, in a sample of 531 Italian consumers (collected by convenience sampling), through offline and online survey platforms. K-means cluster analysis was applied to identify consumer groups using six food choice motivation categories (health, emotional, economic and availability, social and cultural, environmental and political, and marketing and commercial). The results suggest that the strongest determinants for the food choices of Italian consumers are Environmental factors and Health. Two consumer profiles were identified through the segmentation analysis: Emotional eating and Health-driven consumers. The respondents were found to have a good awareness of what comprises a healthy diet. There is a potential market for healthy and sustainable food products, especially products with minimal or environmentally friendly packages. Food labels and information strategies could be promoted as tools to assist consumers to make healthy food choices.

## 1. Introduction

The food that we consume affects our future health. Diet-related non-communicable diseases (NCDs), such as obesity, type 2 diabetes, cardiovascular disease, hypertension, stroke, and some types of cancer, have been increasingly causing health problems in both developing and developed countries [1,2]. Policy-makers have been trying to introduce several different tools to encourage populations to consume healthier foods and reduce their intake of unhealthy foods, through initiatives such as nutritional education programs and fiscal programs (i.e., sugar drink taxes), among others. Despite these attempts, obesity has greatly risen in the past two decades, even in countries where the rates have been historically low, such as Italy [3]. 

In Italy, obesity among adults increased from 9% in 2003 to 11% in 2017. Although obesity in adult remained below than the EU average (15%), nearly one in five 15-year-olds in Italy (18%) were overweight or obese in 2013–2014, a share close to the EU average [4]. This raised public policy concern, as excess weight among children and adolescents could affect the population’s health in the long run. In order to design appropriate policy tools to increase healthy eating, the motivation behind food choices should be understood and defined. 

Food choices are complex and are affected by a combination of various factors, including biological determinants (e.g., hunger, appetite, and taste), psychological determinants (e.g., mood, stress, and guilt), physiological determinants (e.g., access, education, and time), social determinants (e.g., culture, family, and peers), and economic determinants (e.g., cost, income, and availability). Attitudes, beliefs, and knowledge about food also have an influence on food choices [5]. However, these factors could affect people differently, depending upon their context, personality, social groups, and socio-cultural position. 

In the literature it has been discussed that health, mood, convenience, price, familiarity, social norms, natural and ethical concerns, and taste are prime issues considered by consumers when making food choices [6,7,8,9,10]. Eertmans et al. (2006) conducted a survey using the food choice questionnaire (FCQ) in different countries and found that in Italy, health and nature content, convenience and mood were the most important issues Italian consumer concerned in making their food choices [11]. Guiné et al. (2019) studied food choice determinants in Mediterranean countries and found that, in Italy, the food choices were influenced by environmental and political motivation, following by health and emotional reasons [12]. 

In order to determine the eating patterns of individuals in relation to their choices, particularly in the Mediterranean region, a large project entitled “Psycho-social motivations associated with food choices and eating practices” (EATMOT) was carried out. In this framework, a questionnaire was developed to define food choices, according to six types of conditioning motivations (health, emotional, economic and availability, social and culture, environment and politics, and marketing and commercial) [12]. This study was part of the project and its purpose was twofold: (1) To evaluate the food choice motivations in a sample in Italy; and (2) to segment consumer groups and provide consumer profiling, according to their food choice motivations.

## 2. Materials and Methods

### 2.1. Questionnaire

A questionnaire was developed specifically for the EATMOT project by the Center for Education, Technology and Health Studies (CI & DETS Research Center) in Portugal [13]. The questionnaire was prepared in English, then translated into Portuguese for the pre-test and validation of the questionnaire before the actual survey was carried out in 16 countries. The initial scale validity and internal reliability of the questionnaire were assessed only in Portugal (i.e., for the Portuguese version; see details in [13]). After validation, the questionnaire was modified and subsequently translated into English. The final version of the questionnaire was translated into Italian, following a back-translation methodology for validation [14]. During the translation process, the questions were slightly adjusted, in order to be more coherent with Italian culture, while the original meanings were retained. The questionnaire structure included five sections: Part 1—Socio-demographic data; Part 2—Anthropometric data and behavioral- and health-related elements; Part 3—Attitudes relating to healthy food; Part 4—Sources of information about a healthy diet; and Part 5—Food choice motivations (M1: Healthy motivations, M2: Emotional motivations, M3: Economic and availability motivations, M4: Social and cultural motivations, M5: Environmental and political motivations, and M6: Marketing and commercial motivations). The individual items and type of scale for all measures are provided in supplementary materials (Table S1).

The questionnaire comprised both closed- and open-ended questions. In the perception of healthy eating and the food choice motivation sections, respondents were asked to give their opinion toward statements according to a 5-point Likert-like scale, ranging from 1 (Strongly disagree) to 5 (Strongly agree); while in the sources of information section, participants were asked to indicate the frequency at which they found information about healthy diets from different sources, on a scale from 1 (Never) to 5 (Always).

### 2.2. Data Collection

The questionnaire was administered both offline and online (Google forms) using a convenience sample of the Italian population through the personal connections of the authors. The online survey link was distributed through personal emails. Offline data collection was conducted in the North and Central parts of Italy. The interviews were carried out face-to-face with randomly selected consumers in different parts of the town (e.g., grocery stores and supermarkets) by experienced researchers/graduate students under the supervision of the authors of this paper. Data was collected between January and September 2017. The target respondents were adults aged over 18 years old, who voluntarily provided their consent to participate in the study. 

All ethical procedures were strictly followed when designing and applying the questionnaire, and it was ensured that the data provided were kept strictly confidential (i.e., such that no individual response could ever be associated with the respondent). The study was conducted in accordance with the Declaration of Helsinki and the protocol was approved by the Ethics Committee of Polytechnic Institute of Viseu (reference nº 04/2017); furthermore, national and international protocols for research on humans were followed.

In total, 585 individuals participated in the survey. Through the validation process (i.e., elimination of incomplete questionnaires, leaving out outliers, and replacing invalid values with the mean), 531 questionnaires were considered valid and used in data analysis phase.

### 2.3. Data Analysis

Data were analyzed through both univariate and multivariate techniques using the IBM SPSS 26.0 software. A basic descriptive approach was used to describe Italian consumer characteristics, in terms of socio-demographics, anthropometrics, health-related behaviors, and information sources about healthy diets, including perceptions about healthy eating. Body mass index (BMI) was calculated using self-reported height (m) and weight (kg) data. The BMI results were classified according to International Classification Standards [15], as follows: underweight (BMI < 18.50 kg/m^2^), normal weight (18.50 ≤ BMI ≤ 24.99 kg/m^2^), overweight (25.00 ≤ BMI ≤ 29.99 kg/m^2^), and obese (BMI ≥ 30.00 kg/m^2^).

In the food choice motivations section, the median, mean, and standard deviation (SD) values of each item were calculated. Note that, to calculate the global scores, the inverted scores of M1.5 (“There are some foods that I consume regularly even if they may raise my cholesterol”), M1.9 (“There are some foods that I consume regularly even if they may raise my blood glycaemia”), M4.5 (“I prefer to eat alone”), M6.1 (“When I buy food I usually do not care about the marketing campaigns happening in the shop”), and M6.4 (“When I go shopping I prefer to read food labels instead of believing in advertising campaigns”) were used, as they were negative questions (according to the motivations). Hence, the higher the global score, the stronger the influence of the motivations on food choices.

Consumer groups were identified using data of the food choice motivations section. Cronbach’s alpha was used to test the internal validity of the 49 food motivation items (Cronbach’s α = 0.755). Then, all 49 items from the six food choice motivations were used for consumer segmentation (10 healthy items, 9 emotional items, 7 economic and availability items, 9 social and cultural items, 7 environmental and political items, and 7 marketing and commercial items). First, Hierarchical cluster analysis (HCA) with Squared Euclidean distances and Ward’s method was applied to the items, in order to define the optimum number of clusters. The agglomeration schedule suggested that 2 clusters were suitable for the collected data. Then, K-mean cluster analysis was applied to identify the final clusters. Finally, the resulting clusters were evaluated, according to socio-demographics, anthropometrics, health-related behaviors, information sources, and perception about healthy eating, using the Pearson Chi-square, Student t-tests, and Mann–Whitney U test for independent samples. 

## 3. Results

### 3.1. Sample Characteristics

#### 3.1.1. Socio-Demographic and Anthropometric Data and Behavioral and Health Aspects

The sample included 531 Italian participants, of whom 65% were female. Their age ranged from 18 to 75 years, with a large group of respondents aged between 35 and 44 years old (27.7%), followed by respondents aged between 25 and 34 (23.9%). The average age was 42 years (SD = 13.47). The majority of respondents were higher educated and held a university degree (50%), while 45% possessed a secondary school diploma and around 5% of respondents had completed primary school. In terms of residences, 61% of the respondents lived in urban areas, 29% lived in suburban areas, and 10% lived in rural areas. Considering their civil status, 66% of respondents were married/living together, while 26% were single, 6% were divorced or separated, and 2% were widowed. The majority of respondents were employed (56%), while 19% were housewives, 9% were retired, 6% were unemployed, 6% were students, and 4% were working students. Most respondents were responsible for buying food for their household (85%).

Based on the self-reported weight and height, the majority of respondents were of normal weight (63.8%), whereas overweight and obese individuals comprised 24.5% and 5.8% of the study sample, respectively. Two hundred responders (37.7%) described themselves as being physically active, 54.8% of the sample declared having a healthy diet, and 76% of responders were not dieting or following a particular dietary regimen. A total of 72.3% of participants declared not having chronic diseases, while only a few suffered from allergies and/or intolerances (16.6%) or experienced eating disorders (9.2%).

#### 3.1.2. Information Sources

Respondents were asked to indicate the frequency at which they found information about eating a healthy diet from different sources of information. They frequently used the internet and magazines, books and newspapers, and sometimes family or friends, television, and doctors. They found information at school or on the radio sporadically (Table 1).

### 3.2. Perceptions about Healthy Eating

**Figure 1:**
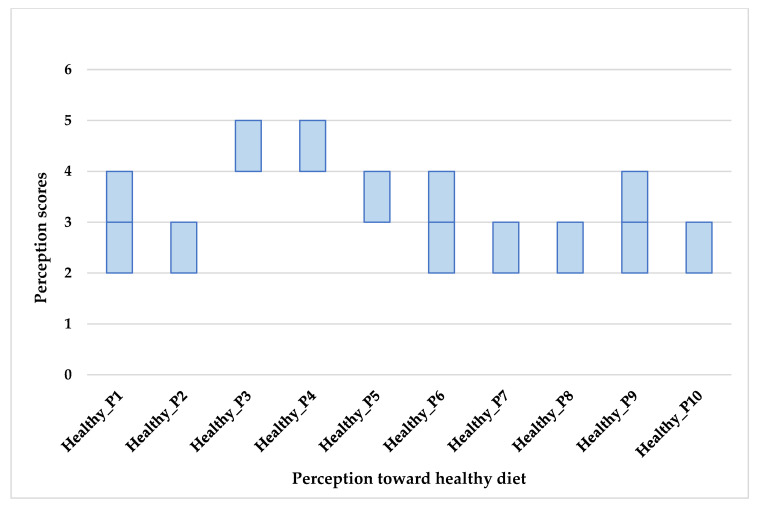
Box plot showing perception scores toward healthy diet (1 = strongly disagree, 2 = disagree, 3 = neither agree nor disagree, 4 = agree, 5 = strongly agree).

The median and average scores of the respondent’s perceptions about healthy eating are displayed in Table 2 and Figure 1. Almost all respondents strongly agreed that a healthy diet should be balanced, varied, complete, and should include fruit and vegetables. They also agreed that it is important to eat everything, although in small quantities. Disagreement was observed for inverted questions related to totally avoiding sugary and fatty products and having cravings for sweets, for some people. For questions related to the price of a healthy diet, the value of tradition for healthy patterns, a healthy diet being based on calorie count, or organically produced foods being healthier than their conventional counterparts, the responders neither agreed nor disagreed; in fact, their scores were quite variable.

We tested the correlations between the different variables recorded in Part 2 of the questionnaire and perceptions toward healthy eating. The following results are those that were correlated and differed among groups at a significance level of 95%. A Kruskal–Wallis test provided strong evidence of a difference (*H* = 9.866, *p* = 0.02, df = 3) among the BMI classes toward the statement “A healthy diet should be balanced, varied and complete”. The Dunn–Bonferroni post-hoc method was carried out. Normal weight respondents rated the highest score for this statement, and there was evidence that it was significantly higher than those of obese (*p* = 0.05) and underweight (*p* = 0.015) respondents, which indicates that this statement could be key to staying healthy and maintaining a normal weight. Underweight respondents rated the statement “We can eat everything, as long as it is in small quantities” significantly lower than normal weight (*p* = 0.013), overweight (*p* = 0.007), and obese (*p* = 0.002) respondents. Underweight respondents also rated the statement “We should never consume fat products” significantly lower than overweight (*p* = 0.028) and obese (*p* = 0.008) respondents. These results meant that underweight respondents were very concerned about the types of food they consumed, but they disagreed that fatty products should be avoided, while overweight and obese respondents were very concerned about consuming fatty products.

The Mann–Whitney U-test was used to assess the following relations. Respondents who stated that they frequently/always followed a healthy diet scored the statements “Fruit and vegetables are very important to healthy eating” (*z* = −2.998, *p* = 0.003) and “A healthy diet should be balanced, varied and complete” (z = −3.193, *p* = 0.001) significantly higher than those who reported that they did not follow a healthy diet. Respondents who stated that they moderately/intensively did physical activities scored the statement “A healthy diet should be balanced, varied and complete” significantly higher than those who did not (*z* = −2.597, *p* = 0.009). Respondents who had chronic diseases scored significantly higher than those who had not on the statements “We should never consume sugary products” (*z* = −2.225, *p* = 0.026) and “We should never consume fat products” (*z* = −2.956, *p* = 0.003), indicating that chronic disease affects the perception of a healthy diet. Respondents who had experienced an eating disorder scored the statement “In my opinion, it is strange that some people have cravings for sweets” significantly lower than those who had never experienced one (*z* = −2.088, *p* = 0.037), showing that, for those who had ever experienced an eating disorder, cravings for sweets were normal. Respondents who followed a voluntary food regimen rated the following statements lower than those who did not follow any food regimen: “We should never consume sugary products” (*z* = −4.931, *p* < 0.001), “We should never consume fat products” (*z* = −3.143, *p* = 0.002), and “I believe that organically produced food is healthier” (*z* = −2.741, *p* = 0.006); while they rated the statement “I believe that tradition is very important to a healthy diet” significantly higher than those who did not follow any food regimen (*z* = −3.432, *p* = 0.001).

In summary, the statistical analysis demonstrated that BMI class and being active significantly affect the perception of a healthy diet as balanced, varied, and complete. Having a chronic disease significantly affects perceptions related to avoiding fatty and sweet products. Respondents who followed a voluntary food regimen had significantly different perceptions about a healthy diet than those who did not follow any regimen, regarding fat and sugar consumption, the role of tradition, and the healthiness of organically produced foods.

### 3.3. Food Motivations

**Figure 2:**
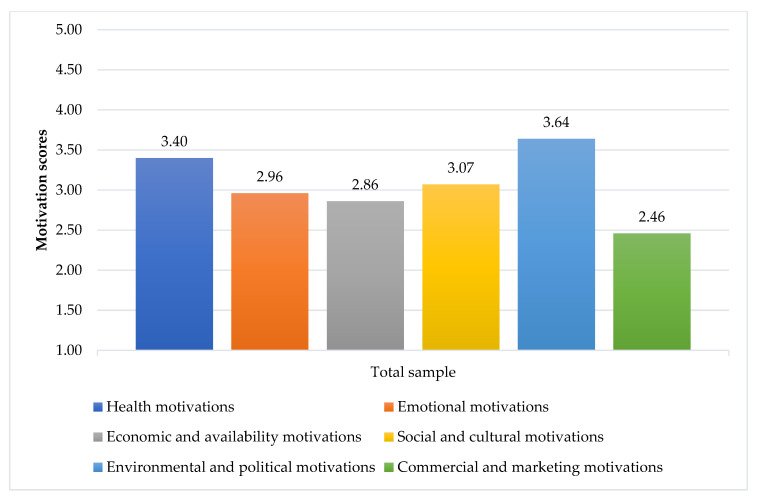
Average motivation scores of total sample (1 = strongly disagree, 2 = disagree, 3 = neither agree nor disagree, 4 = agree, 5 = strongly agree).

The items associated with food choice motivations are shown in Figure 2 and Table 3. Environmental and political motivations (mean = 3.64, SD = 0.57), as well as health (mean = 3.4, SD = 0.46), were the strongest determinants (see Figure 2); while social and cultural (mean = 3.07, SD = 0.36), emotional (mean = 2.96, SD = 0.67), and economic and availability (mean = 2.86, SD = 0.51) motivations were less considered by respondents. Marketing and Commercial motivations were considered the least important drivers of food choices (mean = 2.46, SD = 0.53).

Twenty-three out of the 49 items had a median equal to 4 (median respondents agreed with the statements; see Table 3). It is worth noting that the non-inverted scores of statements M1.5, M1.9, M6.1, and M6.4 (negative statements, according to their global motivations) are shown here as well, as they were actually rated as 4 or above (i.e., agree). Respondents agreed about the social nature of meals (“Meals are a time of fellowship and pleasure”). They preferred to read food labels, instead of believing in marketing and commercial (“When I go shopping I prefer to read food labels instead of believing in advertising campaigns”). They also cared about the quality of their diet, in order to stay healthy (“It is important for me to eat food that keeps me healthy”), and about environmental sustainability (“When I cook I have in mind the quantities to avoid food waste”, “It is important to me that the food I eat is prepared/packed in an environmental friendly way”). Overall, the respondents were very concerned about the environmental and health aspects of their food choices; nevertheless, they also considered emotional (“Food makes me feel good”), economic (“I usually choose food that has a good quality/price ratio”), and social (“I eat more than usual when I have company”) aspects of food. Although the health aspect was crucial for them, they also regularly consumed some foods that may raise their cholesterol and blood glycemia (“There are some foods that I consume regularly, even if they may raise my cholesterol”, “There are some foods that I consume regularly, even if they may raise my blood glycaemia”).

### 3.4. Consumer Segmentation

**Figure 3:**
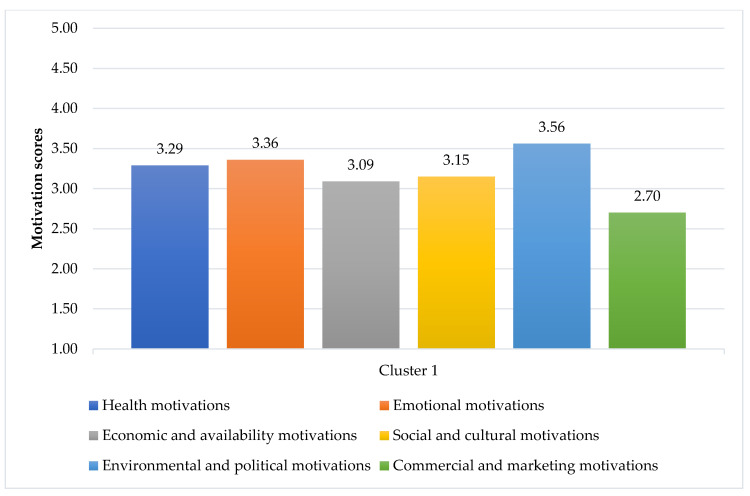
Average motivation scores of Cluster 1 (1 = strongly disagree, 2 = disagree, 3 = neither agree nor disagree, 4 = agree, 5 = strongly agree).

**Figure 4:**
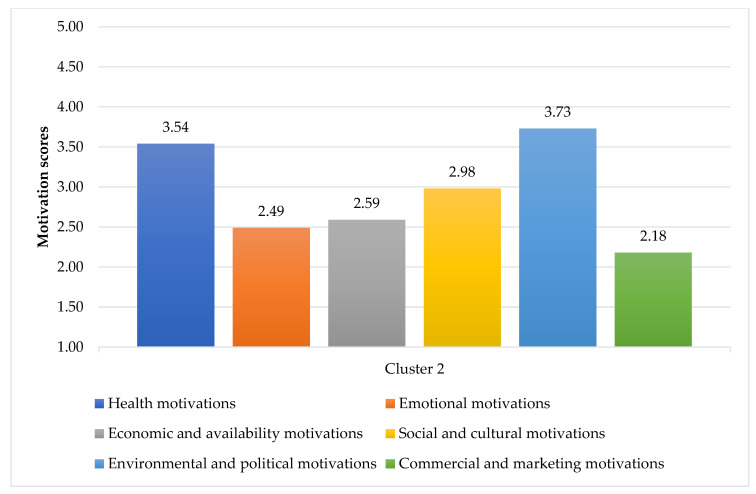
Average motivation scores of Cluster 2 (1 = strongly disagree, 2 = disagree, 3 = neither agree nor disagree, 4 = agree, 5 = strongly agree).

The items associated with the food choice motivations and results from the cluster analysis are shown in Figure 3 and Figure 4. Two clusters were identified: Cluster 1 “Emotional eating consumers” and Cluster 2 “Health-driven consumers”. Regarding the most and least important motivations, both clusters had the same idea: “Environmental and Policy motivations” were the most important, while “Marketing and Commercial motivations” were the least considered. Details of the scores of each item for each motivation, including the statistical difference between Clusters, are given in Appendix A (Table A1).

The first cluster accounted for 54.24% (288 persons) of the total sample and was described as “Emotional eating consumers” (Figure 3). Besides “Environmental and Policy motivations” (mean = 3.56, SD = 0.52), “Emotional motivations” (mean = 3.36, SD = 0.52) were very important for this cluster, as they scored most emotional items higher than the respondents in Cluster 2 (*t* = 19.529, *p* < 0.001). Food helped them to cope with stress, made them feel good, and served as their emotional consolation. In addition, they tended to emotionally eat, as they ate more when they felt lonely or had nothing to do, including craving sweets when they were depressed. They also consumed food to either keep them alert or relax. “Health motivations” (mean = 3.29, SD = 0.46) were the third most important for them; however, they scored most items in this category lower than respondents in Cluster 2, while they scored higher regarding consuming some foods regularly, even if they may raise their cholesterol or blood glycaemia (inverted scores). They also cared for “Social and cultural motivations” (mean = 3.15, SD = 0.37), “Economic and availability motivations” (mean = 3.09, SD = 0.44), and “Marketing and commercial motivations” (mean = 2.70, SD = 0.44) more than respondents in Cluster 2 (*t* = 5.391, *p* < 0.001; *t* = 13.092, *p* < 0.001; and *t* = 12.998, *p* < 0.001, respectively).

The second cluster was named “Health-Driven consumers”, which accounted for 45.76% (243 persons) of the total sample. While “Environmental and Policy motivations” (mean = 3.73, SD = 0.61) were the most important drivers for them, “Health motivations” (mean = 3.54, SD = 0.43) were also highly considered. They agreed that they usually followed a healthy and balanced diet, it was important for them to eat foods that kept them healthy, and they tried to eat foods that did not contain additives. In addition, considering environmental motivations, they preferred to eat food that was prepared/packaged in an environmentally friendly way, and they were concerned about food waste and the reduction of food packages, differing from the respondents in Cluster 1. They cared for the environment more than the respondents in Cluster 1 (*t* = −3.500, *p* = 0.001). Besides those two motivations, “Social and cultural motivations” (mean = 2.98, SD = 0.34), “Economic and availability motivations” (mean = 2.59, SD = 0.44), “Emotional motivations” (mean = 2.49, SD = 0.50), and “Marketing and commercial motivations” (mean = 2.18, SD = 0.49) were less concerned by them.

### 3.5. Consumer Profiling

In order to understand the differences between the two segments, Pearson Chi-square, Student T-test, and Mann–Whitney U-test were performed on their demographic and anthropometric data and behavioral and health aspects. The results revealed that age, life environment, behavior, and health-related elements could significantly differentiate the segments (Table 4).

The average age (44 years old) of respondents in Cluster 2 (or Health-Driven consumers) was significantly higher than that (40 years old) of Cluster 1 (or Emotional eating consumers; *t* = −3.362, *p* = 0.001). A significantly lower percentage of respondents in Cluster 2 (55%) lived in urban areas than those in Cluster 1 (66%; *χ^2^* = 7.936, *p* =0.019). Consistent with their scores for items in Health motivations, 69% of respondents in Cluster 2 stated that they followed a healthy diet. This was significantly higher than that in Cluster 1 (43%; *χ^2^* = 35.059, *p* < 0.001). The respondents in Cluster 2 (46%) also physically exercised more than respondents in Cluster 1 (31%; *χ^2^* = 12.256, *p* < 0.001). Respondents in Cluster 2 (30%) followed a voluntary food regimen more than those in Cluster 1 (24%; *χ^2^* = 8.639, *p* = 0.003). The clusters were also differentiated—although with lower significance (statistically significant at 0.1 level)—in terms of the following issues: Based on BMI categories, a higher number of respondents in Cluster 2 (68%) had normal weight than in Cluster 1 (61%; *z* = −1.839, *p* = 0.066). However, more respondents in Cluster 2 (31%) had chronic diseases than in Cluster 1 (25%; *χ^2^* = 2.888, *p* = 0.089), likely due to their higher average age. Additionally, the respondents in Cluster 1 (11%) had more experiences with eating disorders than those in Cluster 2 (7%; *χ^2^* = 3.738, *p* = 0.053).

Regarding food motivations, respondents in Cluster 1 agreed with 19 out of 49 items (i.e., median respondents agreed with the statements; see Table 5). The non-inverted scores of statements M6.4, M4.5, M1.5, and M1.9 (negative statements, according to their global motivations) are shown here, as they were actually rated as agree. Respondents agreed that food had emotional value for them (“Food makes me feel good”). They also cared about the health (“It is important for me to eat food that keeps me healthy”) and environmental (“When I cook I have in mind the quantities to avoid food waste”) aspects of food. Although they were concerned about economics (“I usually choose food that has a good quality/price ratio”), they preferred to read food labels, instead of believing in advertisements (“When I go shopping I prefer to read food labels instead of believing in advertising campaigns”). Some preferred to eat alone (“I prefer to eat alone”). In general, the respondents in Cluster 1 concerned many aspects (motivations) of food, compared to respondents in Cluster 2. Emotional motivations seemed to be very important to them (“Food makes me feel good”, “I eat more when I have nothing to do”, “I have more cravings for sweets when I am depressed”, and “Food helps me cope with stress”).

Respondents in Cluster 2 agreed with 15 out of 49 items (i.e., median respondents agreed with the statements; see Table 6). The non-inverted scores of statements M6.1 and M6.4 (negative statements, according to their global motivations) are shown here as well. Respondents cared the most about health (“It is important for me to eat food that keeps me healthy”) and environmental (“When I cook I have in mind the quantities to avoid food waste” and “It is important to me that the food I eat is prepared/packed in an environmental friendly way”) aspects of food. They also agreed with social and cultural aspects (“I choose the foods I eat, because it fits the season”). Similar to the respondents in Cluster 1, they preferred to read labels, rather than believing in commercial advertisements (“When I go shopping I prefer to read food labels instead of believing in advertising campaigns”). Generally, the respondents in Cluster 2 were more concerned about health aspects than respondents in Cluster 1 (“It is important for me to eat food that keeps me healthy”, “Usually I follow a healthy and balanced diet”, “It is important for me that my daily diet contains a lot of vitamins and minerals”, and “I try to eat foods that do not contain additives”).

Regarding perceptions about healthy eating, there were also differences on perception about healthy diet between the two clusters (Table 7). Cluster 1 agreed significantly more than cluster 2 with the importance of eating everything (although in small quantities), with the role of tradition in a healthy diet, and that a healthy diet is not cheap. Differences between the clusters about totally avoiding sugary and fatty products were significant at the 0.10 level.

**Figure 5:**
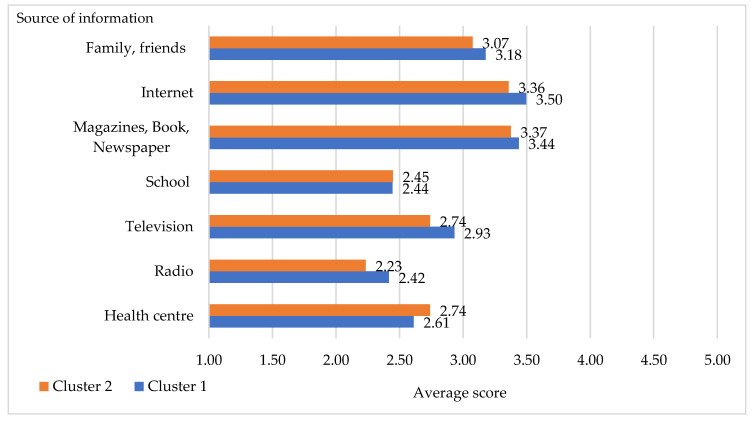
Average scores of information sources (1 = never, 2 = sporadically, 3 = sometimes, 4 = frequently, 5 = always).

The frequency of finding information about eating a healthy diet from different sources of information is shown in Figure 5. Respondents in Cluster 2 used “specialized” sources, such as schools, health centers, hospitals, and family doctors (GP) more than respondents in Cluster 1. On the contrary, respondents in Cluster 1 used mass media—both traditional (radio and television) and internet—as well as books, magazines, and word-of-mouth between family and friends more than respondents in Cluster 2. All these sources, moreover, are cheaper than consulting experts (i.e., doctors).

In order to understand the differences between the information sources used by the two segments, a Mann–Whitney U-test was performed (Table 8). The results revealed that trust in TV and Radio significantly differentiated the segments. The respondents in Cluster 1 had more experience with using Radio and Television for information about healthy eating than those of Cluster 2.

## 4. Discussion

In this study, we investigated the motivations behind food choices in Italy and segmented the surveyed consumers to provide recommendations on effective tools to encourage healthy food choices. The main results indicated that some factors influenced consumer food choices more than others; for instance, in line with the previous literature [11,14,16,17,18,19], “Environmental and Political” and “Health” motivations were the most important determinants of food choices for Italian consumers, while “Marketing and Commercial” motivations were of the least concern [20].

When we looked at individual items, the highest rated items were as follows: Respondents mostly agreed that meals are linked to companionship and pleasure. This might be explained by the Italian tradition that meal time is a time to spend with family and friends [21]. They also stated that they preferred to read food labels when they shop for food, instead of believing in marketing campaigns. It was also highly important for them to eat food that keeps them healthy. Food waste was also highly considered by respondents when they prepare food, which was consistent with the results of Bravia et al. (2020): That Italian consumers tend to be proactive in planning their food purchases and checking ‘use by’ and ‘best before’ dates of food products, such that they can reduce their household food waste [22]. It has also been mentioned, in a report of the European Union, that trying to reduce waste was the number one action Italians carried out to address the issue of climate change [19].

Two consumer clusters were identified, according to the six analyzed food motivations: (1) “Emotional eating consumers”, composed of respondents that were driven by their emotions; and (2) “Health-driven consumers” composed of those that based their choices on the health aspect of food. Although both clusters were primarily concerned with “Environmental and Political” motivations, when it came to food choice, their second-most important motivations differed; namely, “Emotional” and “Health”, respectively. 

The food choices of Emotional eating consumers were affected by psychological factors (e.g., mood). However, they also paid attention to health, food waste, food labels, and economic and availability factors of the product. Basically, they considered more aspects than Health-driven consumers. However, their choices could be highly affected by their mood or emotional state, as they mentioned that food made them feel good, that they ate more when they had nothing to do, that they had more cravings for sweets when they were depressed, and that food helped them to cope with stress. Their average age was lower than that of Health-driven consumers. Our results were in line with those of Cardoso et al. (2020), who stated that young adults had eating behaviors which were more conditioned by emotional motivations (e.g., to fight loneliness or boredom), compared to senior adults and elderly people [23]. Moreover, young adults have also been reported to link eating food with emotional consolation; for example, to help deal with stress and negative moods [24]. In addition, Emotional eating consumers had more experiences with eating disorders than Health-driven consumers, as they tended to exhibit emotional eating behaviors. Emotional eating consumers also agreed that there was some food that they consumed regularly, even if it may raise their cholesterol/blood glycaemia.

The food choices of Health-driven consumers were mainly driven by health-related aspects, seasonal availability, and label information. The relevance of sustainable consumption movements was also highlighted in this cluster. Besides avoiding creating food waste, similarly to Emotional eating consumers, they also believed that food should be prepared/packaged in an environmentally friendly manner, using minimal packaging. For them, it was also important that the foods they consume came from countries where human rights are not violated. When we looked at their profiles, the average age of Health-driven consumers was significantly higher than Emotional eating consumers. As a consequence, they had more chronic diseases than Emotional eating consumers. Health-driven consumers also stated that they followed a healthy diet and/or voluntary food regimen and exercised more than Emotional eating consumers. This is consistent with the fact that elderly individuals tend to eat more fruit and vegetables and are usually more adherent to healthy diets than young adults [25]. The fact that they have more chronic diseases may drive them to adhere to dietary recommendations and to be active. As a consequence, they tended to have a normal weight more than Emotional eating consumers.

Regarding perceptions related to healthy eating, most respondents perceived that a healthy diet should be balanced, varied, and complete and should include fruit and vegetables, which are aspects in accordance with suggestions for healthy eating by nutritionists [26,27,28,29]. This demonstrated that they were mostly aware about what healthy foods are. Nevertheless, only half of them stated that they followed a healthy diet. Hence, there existed a gap between declarative knowledge and behaviors. Several studies have confirmed that healthy eating knowledge is a significant predictor of both future knowledge and behavior [30,31,32,33]; however, knowledge alone is not sufficient to change the food behaviors of consumers, as such behaviors can also be influenced by personal, intra-individual, and environmental factors, including motivations [34,35,36]. The results of our cluster analysis underline that one such factor could be “Emotional” motivations; at least, for Emotional eating consumers. In addition, unconscious motivations and the link between nutritional knowledge, emotions, and food choice should be further investigated.

Most respondents also agreed that they could eat everything, as long as it is in a small quantity. In addition, they disagreed about the “total avoidance of sugary and fatty products”. This may be because they believed that small quantities and variety are key to a healthy diet, as suggested by the Italian Food Dietary Guideline [37]. Moreover, they may have taken into account the emotional effect on food choice, as they generally agreed that having a craving for sweets is not unusual. 

Respondents who had a normal body weight, declared to have healthy diet, and were active believed that a healthy diet should be balanced, varied, and complete. Overweight and obese respondents were more concerned about the consumption of fatty products than other groups (i.e., agreeing more that we should avoid consuming fatty products). Respondents who had a chronic disease perceived that they should avoid fatty and sweet products, as suggested in the various guidelines for the treatment of heart disease and for preventing dietary diseases in the general population (see, e.g., the Diet and Lifestyle Recommendations by the American Heart Association) [38]. 

In addition, following a voluntary food regimen led respondents to perceive healthy eating differently than those who did not follow any voluntary food regimen, with respect to fat and sugar consumption, the role of tradition, and the healthiness of organically produced foods. This might be because individuals who follow a particular diet are usually more attentive to their diet and are more informed about their diet, such that they are more aware of what should be limited (e.g., fat and sugar). They also considered that organic products are produced with more respect for the environment [39]. Especially in Italy, organic products are considered not only environmentally friendly but healthy; although, from a nutritional point of view, organic products are not considered to differ much from non-organic ones [39,40,41].

Concerning the sources of information used, our sample was well-informed and took information from various sources. The internet was the main source of information about healthy eating, followed by magazines, books, and newspapers. These answers were consistent with the emerging informative use of the Internet (i.e., reading online newspapers, documenting health, collecting information on products), although the Italian people are still in the last position in Europe, in terms of informative usage of the Internet, at present [42]. The internet also consists of the use of social networks. The use of social networks has been shown to change the way that consumers search for information and select products; they are becoming prominent sources of information, including for food choices (see [43,44], for example). From this perspective, social media can influence information strategies in two ways: Reducing the cost of releasing information, compared to that of traditional mass media (i.e., television or radio), and making specific consumer groups more easily targeted.

Offline and online word-of-mouth and social media, however, can be dangerous in transmitting misinformation; especially in the food sector [45,46]. Indeed, the recent study of Castellini et al. (2020) found that around half of Italians (48%) admitted that they had believed in a news story about the food sector that turned out to be false at least a few times in the last year, while a third of those (37%) had shared it on social media, thus contributing to the unstoppable spread of “food fake news” [46]. An even more interesting fact is that this phenomenon occurs in all social and educational classes. In particular, individuals who believe in such misinformation are psychologically different than consumers who are less persuaded by this kind of news. They are more driven by other motivations, related to familiarity with the product and the mood of the moment, rather than by the evaluation of healthiness of the food. They are more favorably oriented to experimentation with new products and are more predisposed to social influence, being less self-confident [46]. Hence, the Emotional eating consumers could be at risk of being more susceptible to such misinformation. This means that the role of institutions in educating the public is important, and that they should exploit different media forms, in order to aid citizens to be able to distinguish between reliable and non- reliable sources of information and enable them to make well-informed food choices. 

For policy-makers, food labels could be promoted as a tool to assist consumers to make a conscious food choice. Information provision to promote sustainable and healthy diets could be carried out through educational campaigns (e.g., relating to the inclusion of sustainability in dietary guidelines) or improved sustainability labelling on packaging. Social media could be used to change social norms and food culture towards healthy diets and waste reduction, as recent studies have demonstrated that social media information also affects environmental awareness and consumer information and choices relating to sustainable food [44]. Further research is necessary to examine the role of the Internet in food information more closely, as well as the sources which are judged as most reliable by respondents. It will also be interesting, in future research, to deepen the multifaceted relationship between traditional and social media information and healthy or sustainable consumption, in terms of food choices for specific consumer segments. 

For food marketers, there was a clear need, for both clusters, for food products that are healthy and sustainable. Environmentally friendly packaging and human rights in the producing countries were also emphasized by Health-driven consumers. Moreover, information regarding the environment and sustainability on these issues (e.g., product labelling) should be provided, in order to increase the purchase of such products [47].

The limitations of this research were as follows: There was a higher proportion of female participants and the sample had higher education, on average, than the Italian census. BMI values were calculated from self-reported height and weight and, therefore, the results should be interpreted with care. We also did not ask respondents about their dietary habits or their consumption of some food products, such that we could not compare their statements (e.g., following healthy diet) and (reported) consumption behavior. Future research should cover this aspect.

## 5. Conclusions

In conclusion, the respondents had a good awareness of what a healthy diet consists of. They mostly found information online or in newspapers and books, as well as through talking with friends and families. The strongest determinants for their food choice were Environmental and Health factors. The less influential reasons were those related to Marketing and Commercial. The clustering analysis resulted in two consumer segments: Emotional eating consumers and Health-driven consumers. Both segments considered Environmental and Political motivations as the most important issues. Nevertheless, their second-most important motivations divided them, as the Emotional eating consumers were more influenced by their emotions, while Health-driven consumers were more concerned with the health aspects of food. Emotional eating consumers were younger, while Health-driven consumers had more normal weight and stated that they followed a healthy diet and/or voluntary food regimen and exercised more than Emotional eating consumers. Food labels were used by respondents as an important tool when making food choices. Food waste and food packaging were issues also concerned by most respondents.

## Figures and Tables

**Table 1:** Sources of information for the total sample (*n* = 531).

Source of Information	Median	Mean	SD
Internet	4	3.43	1.07
Magazines, books, newspapers	4	3.41	0.93
Family, friends	3	3.13	0.87
Television	3	2.84	1.03
Health center, hospitals, family doctor (General Practitioner, GP)	3	2.67	1.11
School	2	2.45	1.15
Radio	2	2.33	1.01

Note: Respondents were asked to indicate the frequency at which they found information about eating a healthy diet, on the following scale: 1 = never, 2 = sporadically, 3 = sometimes, 4 = frequently, 5 = always.

**Table 2:** Perceptions about healthy eating (*n* = 531).

	Statement	Median	Mean	SD
P.4	A healthy diet should be balanced, varied and complete	5	4.66	0.73
P.3	Fruit and vegetables are very important to healthy eating	5	4.65	0.70
P.5	We can eat everything, as long as it is in small quantities	4	3.44	1.03
P.9	I believe that organically produced food is healthier	3	3.20	1.11
P.1	A healthy diet is based on calorie count	3	2.95	1.02
P.8	I believe that tradition is very important to a healthy diet	3	2.82	0.95
P.6	I believe that a healthy diet is not cheap	3	2.70	1.13
P.2	We should never consume sugary products	2	2.55	1.11
P.10	We should never consume fat products	2	2.38	0.97
P.7	In my opinion, it is strange that some people have cravings for sweets	2	2.25	0.92

Note: Respondents were asked to indicate their opinion on the statements, based on a 5-point semantic scale: 1 = strongly disagree, 2 = disagree, 3 = neither agree nor disagree, 4 = agree, 5 = strongly agree.

**Table 3:** The most important food choice motivations for the total sample (*n* = 531).

	Statement	Mean	SD
M4.1	Meals are a time of fellowship and pleasure	4.34	0.68
M6.4	When I go shopping, I prefer to read food labels instead of believing in advertising campaigns	4.05	0.86
M1.8	It is important for me to eat food that keeps me healthy	4.02	0.76
M5.2	When I cook, I have in mind the quantities to avoid food waste	4.00	0.79
M5.1	It is important to me that the food I eat is prepared/packed in an environmentally friendly way	3.85	0.78
M4.6	I choose the foods I eat because it fits the season	3.82	0.83
M2.5	Food makes me feel good	3.81	0.85
M3.1	I usually choose food that has a good quality/price ratio	3.81	0.76
M5.4	I prefer to eat food that has been produced in a way that animals’ rights have been respected	3.81	0.90
M4.2	I eat more than usual when I have company	3.79	0.90
M1.4	It is important for me that my daily diet contains a lot of vitamins and minerals	3.77	0.75
M4.8	I like to try new foods to which I am not accustomed	3.75	1.01
M5.5	I choose foods that have been produced in countries where human rights are not violated	3.71	0.92
M1.1	I am very concerned about the hygiene and safety of the food I eat	3.69	0.88
M3.4	I buy fresh vegetables to cook myself more often than frozen	3.66	1.10
M1.3	Usually, I follow a healthy and balanced diet	3.63	0.87
M1.10	I avoid foods with genetically modified organisms	3.60	1.11
M5.7	I prefer to buy foods that comply with policies of minimal usage of packaging	3.59	0.91
M5.3	It is important to me that the food I eat comes from my own country	3.56	1.03
M1.6	I try to eat foods that do not contain additives	3.50	0.96
M6.1	When I buy food, I usually do not care about the marketing campaigns happening in the shop	3.43	0.93
M1.5	There are some foods that I consume regularly, even if they may raise my cholesterol	3.35	0.95
M1.9	There are some foods that I consume regularly, even if they may raise my blood glycaemia	3.32	0.93

Note: Respondents were asked to indicate their opinion on the statements based on a 5-point semantic scale: 1 = strongly disagree, 2 = disagree, 3 = neither agree nor disagree, 4 = agree, 5 = strongly agree. Median scores of all items were equal to 4 (agree).

**Table 4:** Socio-demographic, anthropometric data, behavioral- and health-related elements of clusters.

Item	Frequency (%)			*p*-Value
	Total (*n* = 531)	Cluster 1 (*n* = 288)	Cluster 2 (*n* = 243)	
Age mean (SD)	42.09 (13.47)	40.30 (13.12)	44.21 (13.60)	0.001 ^a^
Age range	35–44	35–44	35–44	<0.001 ^b^
18–24	45 (8.5%)	29 (10.1%)	16 (6.6%)	
25–34	127 (23.9%)	78 (27.1%)	49 (20.2%)	
35–44	147 (27.7%)	88 (30.6%)	59 (24.3%)	
45–54	109 (20.5%)	48 (16.7%)	61 (25.1%)	
≥55	103 (19.4%)	45 (15.6%)	58 (23.9%)	
Gender	Female	Female	Female	0.180
Female	346 (65.2%)	195 (67.7%)	151 (62.1%)	
Male	185 (34.8%)	93 (32.3%)	92 (37.9%)	
Education (Highest level) (Median)	University	University	Secondary school	0.347
Primary school	26 (4.9%)	14 (4.9%)	12 (4.9%)	
Secondary school	238 (44.8%)	121 (42%)	117 (48.1%)	
University	267 (50.3%)	153 (53.1%)	114 (46.9%)	
Life environment (Median)	Urban	Urban	Urban	0.019
Rural	56 (10.5%)	31 (10.8%)	25 (10.3%)	
Urban	323 (60.8%)	189 (65.6%)	134 (55.1%)	
Suburban	152 (28.6%)	68 (23.6%)	84 (34.6%)	
Civil status (Median)	Married	Married	Married	0.278
Single	139 (26.2%)	82 (28.5%)	57 (23.5%)	
Married/Living together	351 (66.1%)	187 (64.9%)	164 (67.5%)	
Divorced/Separated	31 (5.8%)	16 (5.6%)	15 (6.2%)	
Widow	10 (1.9%)	3 (1.0%)	7 (2.9%)	
Professional activity (Median)	Employed	Employed	Employed	0.106
Employed	299 (56.3%)	165 (57.3%)	134 (55.1%)	
Unemployed	30 (5.6%)	12 (4.2%)	18 (7.4%)	
Student	34 (6.4%)	24 (8.3%)	10 (4.1%)	
Retired	47 (8.9%)	20 (6.9%)	27 (11.1%)	
Student worker	20 (3.8%)	12 (4.2%)	8 (3.3%)	
Housewife	101 (19.0%)	55 (19.1%)	46 (18.9%)	
Responsible for food buying	Responsible	Responsible	Responsible	0.561
Responsible	451 (84.9%)	247 (85.8%)	204 (84%)	
Not responsible	80 (15.1%)	41 (14.2%)	39 (16%)	
BMI mean (SD)	23.65 (3.77)	23.88 (3.96)	23.36 (3.51)	0.110 ^a^
BMI categories (Median)	Normal weight	Normal weight	Normal weight	0.066 ^b^
Underweight (BMI < 18.50)	31 (5.8%)	16 (5.6%)	15 (6.2%)	
Normal weight (18.50 ≤ BMI ≤ 24.99)	339 (63.8%)	175 (60.8%)	164 (67.5%)	
Overweight (25.00 ≤ BMI ≤ 29.99)	130 (24.5%)	76 (26.4%)	54 (22.2%)	
Obese (BMI ≥ 30.00)	31 (5.8%)	21 (7.3%)	10 (4.1%)	
Physical exercise	Not enough	Not enough	Not enough	<0.001
Not enough physical exercise	331 (62.3%)	199 (69.1%)	132 (54.3%)	
Enough physical exercise	200 (37.7%)	89 (30.9%)	111 (45.7%)	
Healthy diet	Healthy diet	Unhealthy diet	Healthy diet	<0.001
Unhealthy diet	240 (45.2%)	164 (56.9%)	76 (31.3%)	
Healthy diet	291 (54.8%)	124 (43.1%)	167 (68.7%)	
Voluntary eating regimen	No	No	No	0.003
No voluntary regimen	401 (75.5%)	232 (80.6%)	169 (69.5%)	
Voluntary regimen	130 (24.5%)	56 (19.4%)	74 (30.5%)	
Chronic disease	No	No	No	0.089
No chronic disease	384 (72.3%)	217 (75.3%)	167 (68.7%)	
Chronic disease	147 (27.7%)	71 (24.7%)	76 (31.3%)	
Allergies/Intolerances	No	No	No	0.268
No allergies/intolerances	443 (83.4%)	245 (85.1%)	198 (81.5%)	
Allergies/intolerances	88 (16.6%)	43 (14.95%)	45 (18.5%)	
Have ever experienced eating disorder	No	No	No	0.053
No	482 (90.8%)	255 (88.5%)	227 (93.4%)	
Yes	49 (9.2%)	33 (11.5%)	16 (6.6%)	

Note: *p*-values were results from Pearson Chi-square, except ^a^, which resulted from a Student’s T-test and ^b^, which resulted from a Mann–Whitney U-Test between the two Clusters.

**Table 5:** The most important food choice motivations for Cluster 1 (*n* = 288).

	Statement	Mean	SD
M2.5	Food makes me feel good	3.99	0.75
M1.8	It is important for me to eat food that keeps me healthy	3.92	0.79
M5.2	When I cook, I have in mind the quantities to avoid food waste	3.91	0.76
M6.4	When I go shopping, I prefer to read food labels instead of believing in advertising campaigns	3.89	0.84
M3.1	I usually choose food that has a good quality/price ratio	3.88	0.70
M4.5	I prefer to eat alone	3.85	0.99
M2.7	I eat more when I have nothing to do	3.76	0.97
M1.4	It is important for me that my daily diet contains a lot of vitamins and minerals	3.72	0.76
M4.6	I choose the foods I eat because it fits the season	3.69	0.81
M5.1	It is important to me that the food I eat is prepared/packed in an environmentally friendly way	3.67	0.75
M5.5	I choose foods that have been produced in countries where human rights are not violated	3.63	0.84
M1.5	There are some foods that I consume regularly, even if they may raise my cholesterol	3.58	0.79
M1.9	There are some foods that I consume regularly, even if they may raise my blood glycaemia	3.55	0.79
M1.10	I avoid foods with genetically modified organisms	3.54	1.05
M2.9	I have more cravings for sweets when I am depressed	3.54	1.08
M3.4	I buy fresh vegetables to cook myself more often than frozen	3.54	1.08
M3.5	I usually buy food that is easy to prepare	3.50	0.85
M2.1	Food helps me cope with stress	3.47	0.84
M1.3	Usually, I follow a healthy and balanced diet	3.44	0.90

Note: Respondents were asked to indicate their opinion on the statements, based on a 5-point semantic scale (1 = strongly disagree, 2 = disagree, 3 = neither agree nor disagree, 4 = agree, 5 = strongly agree). Median scores of all items were equal to 4 (agree).

**Table 6:** The most important food choice motivations for Cluster 2 (*n* = 243)

	Statement	Mean	SD
M1.8	It is important for me to eat food that keeps me healthy	4.14	0.70
M5.2	When I cook, I have in mind the quantities to avoid food waste	4.12	0.82
M5.1	It is important to me that the food I eat is prepared/packed in an environmentally friendly way	4.05	0.77
M4.6	I choose the foods I eat because it fits the season	3.97	0.83
M6.4	When I go shopping, I prefer to read food labels instead of believing in advertising campaigns	4.24	0.84
M1.3	Usually, I follow a healthy and balanced diet	3.86	0.76
M1.4	It is important for me that my daily diet contains a lot of vitamins and minerals	3.83	0.73
M3.4	I buy fresh vegetables to cook myself more often than frozen	3.81	1.11
M5.5	I choose foods that have been produced in countries where human rights are not violated	3.81	1.00
M5.7	I prefer to buy foods that comply with policies of minimal usage of packaging	3.76	0.92
M1.6	I try to eat foods that do not contain additives	3.73	0.89
M3.1	I usually choose food that has a good quality/price ratio	3.72	0.83
M1.10	I avoid foods with genetically modified organisms	3.68	1.17
M2.5	Food makes me feel good	3.60	0.91
M6.1	When I buy food, I usually do not care about the marketing campaigns happening in the shop	3.52	1.04

Note: Respondents were asked to indicate their opinion on the statements, based on a 5-point semantic scale (1 = strongly disagree, 2 = disagree, 3 = neither agree nor disagree, 4 = agree, 5 = strongly agree). Median scores of all items are equal to 4 (agree).

**Table 7:** Differences between clusters on perceptions about healthy eating.

Perception	Cluster 1 (*n* = 288)			Cluster 2 (*n* = 243)			*p*-Value
	Median	Mean	SD	Median	Mean	SD	
P.5 We can eat everything, as long as it is in small quantities	4	3.55	0.95	3	3.32	1.10	0.024
P.6 I believe that a healthy diet is not cheap	3	2.80	1.06	2	2.58	1.20	0.009
P.8 I believe that tradition is very important to a healthy diet	3	2.91	0.98	3	2.70	0.89	0.014
P.2 We should never consume sugary products	2	2.46	1.05	2	2.66	1.17	0.075
P.10 We should never consume fat products	2	2.43	0.92	2	2.32	1.03	0.099

Note: Respondents were asked to indicate their opinion on the statements based on a 5-semantic scale (1 = strongly disagree, 2 = disagree, 3 = neither agree nor disagree, 4 = agree, 5 = strongly agree). The *p*-values are the result of Mann–Whitney U-Test between two clusters.

**Table 8:** Difference in information sources about healthy diets between clusters.

Information Sources	Cluster 1 (*n* = 288)			Cluster 2 (*n* = 243)			*p*-Value
	Median	Mean	SD	Median	Mean	SD	
Radio	3	2.42	1.01	2	2.23	1.00	0.032
Television	3	2.93	0.98	3	2.74	1.09	0.041

Note: Respondents were asked to indicate the frequency at which they found information about eating a healthy diet, on the following scale: 1 = never, 2 = sporadically, 3 = sometimes, 4 = frequently, 5 = always. The *p*-values are the results of Mann–Whitney U-Test between two clusters.

## Data Availability

The datasets generated for this study are available on request to the corresponding author.

## Supplementary Materials

The following are available online at https://www.mdpi.com/2304-8158/10/2/318/s1, Table S1: Overview of measures used in the study

## Appendix A

Food choice motivation of total sample, Cluster 1 and Cluster 2.

**Table A1:** Food choice motivation of total sample, Cluster 1 and Cluster 2.

Statement	Total (*n* = 531)			Cluster 1 (*n* = 288)			Cluster 2 (*n* = 243)			*p*-Value
	Median	Mean	SD	Median	Mean	SD	Median	Mean	SD	
**M1. Healthy Motivations**										
M1.1 I am very concerned about the hygiene and safety of the food I eat	4	3.69	0.88	4	3.66	0.85	4	3.71	0.93	0.347
M1.2 It is important for me that my diet is low in fat	3	3.26	0.88	3	3.28	0.85	3	3.23	0.91	0.422
M1.3 Usually I follow a healthy and balanced diet	4	3.63	0.87	4	3.44	0.90	4	3.86	0.76	<0.001
M1.4 It is important for me that my daily diet contains a lot of vitamins and minerals	4	3.77	0.75	4	3.72	0.76	4	3.83	0.73	0.047
M1.5 There are some foods that I consume regularly, even if they may raise my cholesterol ^a^	2	2.65	0.95	2	2.42	0.79	3	2.93	1.05	<0.001
M1.6 I try to eat foods that do not contain additives	4	3.50	0.96	3	3.30	0.96	4	3.73	0.89	<0.001
M1.7 I avoid eating processed foods, because of their lower nutritional quality	3	3.23	0.98	3	3.14	0.95	3	3.33	1.01	0.024
M1.8 It is important for me to eat food that keeps me healthy	4	4.02	0.76	4	3.92	0.79	4	4.14	0.70	0.001
M1.9 There are some foods that I consume regularly, even if they may raise my blood glycaemia ^a^	2	2.68	0.93	2	2.45	0.79	3	2.94	1.00	<0.001
M1.10 I avoid foods with genetically modified organisms	4	3.60	1.11	4	3.54	1.05	4	3.68	1.17	0.044
**M2. Emotional Motivations**										
M2.1 Food helps me cope with stress	3	2.98	1.05	4	3.47	0.84	2	2.40	0.98	<0.001
M2.2 I usually eat food that helps me control my weight	3	3.04	0.97	3	3.02	0.94	3	3.07	1.01	0.382
M2.3 I often consume foods that keep me awake and alert (such as coffee, coke, energy drinks)	2	2.67	1.20	3	3.05	1.20	2	2.22	1.04	<0.001
M2.4 I often consume foods that helps me relax (e.g., teas, red wine)	3	3.01	1.13	3	3.21	1.08	3	2.78	1.14	<0.001
M2.5 Food makes me feel good	4	3.81	0.85	4	3.99	0.75	4	3.60	0.91	<0.001
M2.6 When I feel lonely, I console myself by eating	2	2.47	1.07	3	3.05	0.98	2	1.70	0.73	<0.001
M2.7 I eat more when I have nothing to do	3	3.20	1.21	4	3.76	0.97	2	2.55	1.13	<0.001
M2.8 For me, food serves as an emotional consolation	2	2.56	1.16	3	3.18	1.03	2	1.82	0.82	<0.001
M2.9 I have more cravings for sweets when I am depressed	3	2.93	1.28	4	3.54	1.08	2	2.20	1.12	<0.001
**M3. Economic and Availability Motivations**										
M3.1 I usually choose food that has a good quality/price ratio	4	3.81	0.76	4	3.88	0.70	4	3.72	0.83	0.034
M3.2 The main reason for choosing a food is its low price	2	1.88	0.88	2	2.18	0.91	1	1.52	0.68	<0.001
M3.3 I choose the food I consume because it is convenient to purchase	2	2.54	1.00	3	1.86	0.94	2	2.17	0.94	<0.001
M3.4 I buy fresh vegetables to cook myself more often than frozen	4	3.66	1.10	4	3.54	1.08	4	3.81	1.11	0.001
M3.5 I usually buy food that is easy to prepare	3	3.11	1.04	4	3.50	0.85	3	2.66	1.06	<0.001
M3.6 I usually buy food that it is on sale	3	3.08	0.97	3	3.43	0.83	3	2.67	0.96	<0.001
M3.7 I prefer to buy food that is ready to eat or pre-cooked	2	1.94	0.94	2	2.24	0.99	1	1.57	0.73	<0.001
**M4. Social and Cultural Motivations**										
M4.1 Meals are a time of fellowship and pleasure	4	4.34	0.68	4	4.36	0.62	4	4.32	0.74	0.881
M4.2 I eat more than usual when I have company	4	3.79	0.90	4	3.82	0.90	4	3.76	0.91	0.482
M4.3 It is important to me that the food I eat is similar to the food I ate when I was a child	2	2.40	0.93	3	2.52	0.92	2	2.24	0.91	<0.001
M4.4 I eat certain foods because other people (my colleagues, friends, family) also eat it	2	2.15	0.93	2	2.43	0.94	2	1.81	0.80	<0.001
M4.5 I prefer to eat alone ^a^	4	3.95	0.97	4	3.85	0.99	4	4.06	0.94	0.009
M4.6 I choose the foods I eat because it fits the season	4	3.82	0.83	4	3.69	0.81	4	3.97	0.83	<0.001
M4.7 I eat certain foods because I am expected to eat them	2	1.85	0.90	2	2.05	0.96	1	1.61	0.75	<0.001
M4.8 I like to try new foods to which I am not accustomed	4	3.75	1.01	4	3.75	1.02	4	3.75	0.99	0.925
M4.9 I usually eat food that is trendy	1	1.64	0.76	2	1.89	0.83	1	1.35	0.54	<0.001
**M5. Environment and Political Motivations**										
M5.1 It is important to me that the food I eat is prepared/packed in an environmentally friendly way	4	3.85	0.78	4	3.67	0.75	4	4.05	0.77	<0.001
M5.2 When I cook, I have in mind the quantities to avoid food waste	4	4.00	0.79	4	3.91	0.76	4	4.12	0.82	<0.001
M5.3 It is important to me that the food I eat comes from my own country	4	3.56	1.03	4	3.53	0.98	4	3.58	1.09	0.321
M5.4 I prefer to eat food that has been produced in a way that animals’ rights have been respected	4	3.81	0.90	4	3.79	0.85	4	3.84	0.97	0.310
M5.5 I choose foods that have been produced in countries where human rights are not violated	4	3.71	0.92	4	3.63	0.84	4	3.81	1.00	0.009
M5.6 I avoid going to restaurants that do not have a recovery policy of food surplus	3	2.96	0.84	3	2.94	0.79	3	2.98	0.89	0.626
M5.7 I prefer to buy foods that comply with policies of minimal usage of packaging	4	3.59	0.91	3	3.45	0.87	4	3.76	0.92	<0.001
**M6. Market and Commercials Motivations**										
M6.1 When I buy food, I usually do not care about the marketing campaigns happening in the shop ^a^	2	2.57	0.93	3	2.65	0.83	2	2.48	1.04	0.012
M6.2 I eat what I eat, because I recognize it from advertisements or have seen it on TV	2	1.85	0.81	2	2.09	0.81	1	1.57	0.73	<0.001
M6.3 I usually buy food that spontaneously appeals to me (e.g., situated at eye level, appealing colors, pleasant packaging)	2	2.27	0.97	3	2.63	0.90	2	1.85	0.87	<0.001
M6.4 When I go shopping, I prefer to read food labels instead of believing in advertising campaigns ^a^	2	1.95	0.86	2	2.11	0.84	2	1.76	0.84	<0.001
M6.5 Food advertising campaigns increase my desire to eat certain foods	2	2.47	1.06	3	2.86	0.96	2	2.01	0.98	<0.001
M6.6 Brands are important to me when making food choices	3	2.96	1.10	3	3.14	0.99	3	2.75	1.18	<0.001
M6.7 I try to schedule my food shopping for when I know there are promotions or discounts	3	3.17	1.04	3	3.44	0.93	3	2.84	1.06	<0.001

Note: Respondents were asked to indicate their opinion on the statements based on a 5-point semantic scale (1 = strongly disagree, 2 = disagree, 3 = neither agree nor disagree, 4 = agree, 5 = strongly agree). ^a^ Inverted scale as they were negative questions (according to the motivations), *p*-values are results from Mann–Whitney U-Test between the two clusters.
