# Supplementary material for: Food Choice Determinants and Perceptions of a Healthy Diet among Italian Consumers
Source: Foods. 2021 Feb 3;10(2):318. doi: 10.3390/foods10020318 (PMC7913531; doi:10.3390/foods10020318)
Supplement: Supplementary file 1 [file foods-10-00318-s001.pdf]

Supplementary Material

Table S1. Overview of measures used in the study.

Measures	Item/Statement	Scale
Perception about Healthy Eating	Indicate your opinion about the following statements.	
P.1	A healthy diet is based on calorie count	
P.2	We should never consume sugary products	
P.3	Fruit and vegetables are very important to a practice of a healthy eating	
P.4	A healthy diet should be balanced, varied and complete	5-point scale from "Strongly Disagree" (1) to "Strongly Agree" (5)
P.5	We can eat everything, as long as it is in small quantities	
P.6	I believe that a healthy diet is not cheap	
P.7	In my opinion it is strange that some people have cravings for sweets	
P.8	I believe that tradition is very important to a healthy diet	
P.9	I believe that food produced in a biological way is healthier	
P.10	We should never consume fat products	
Sources of information about healthy diet	Where do you usually find information about eating a healthy diet?<br>Indicate with what frequency you find information about eating a healthy diet.	
I.1	Health centres, hospitals, general practitioner	
I.2	Radio	
I.3	Television	5-point scale from "Never" (1) to "Always" (5)
I.4	School	
I.5	Magazines, books, newspapers	
I.6	Internet	
I.7	Family, friends	
**Food choice motivations**	Indicate your opinion about the following statements.	
Health Motivations		
M1.1	I am very concerned about the hygiene and safety of the food I eat	
M1.2	It is important for me that my diet is low in fat	
M1.3	Usually I follow a healthy and balanced diet	
M1.4	It is important for me that my daily diet contains a lot of vitamins and minerals	5-point scale from "Strongly Disagree" (1) to "Strongly Agree" (5)
M1.5	There are some foods that I consume regularly, even if they may raise my cholesterol	
M1.6	I try to eat foods that do not contain additives	
M1.7	I avoid eating processed foods, because of their lower nutritional quality	
M1.8	It is important for me to eat food that keeps me healthy	
M1.9	There are some foods that I consume regularly, even if they may raise my blood glycaemia	
M1.10	I avoid foods with genetically modified organisms	

Emotional motivations		
M2.1	Food helps me cope with stress	5-point scale from "Strongly Disagree" (1) to "Strongly Agree" (5)
M2.2	I usually eat food that helps me control my weight	
M2.3	I often consume foods that keep me awake and alert (such as coffee, coke, energy drinks)	
M2.4	I often consume foods that helps me relax (such as some teas, red wine)	
M2.5	Food makes me feel good	
M2.6	When I feel lonely, I console myself by eating	
M2.7	I eat more when I have nothing to do	
M2.8	For me, food serves as an emotional consolation	
M2.9	I have more cravings for sweets when I am depressed	
Economic and availability motivations		
M3.1	I usually choose food that has a good quality/price ratio	5-point scale from "Strongly Disagree" (1) to "Strongly Agree" (5)
M3.2	The main reason for choosing a food is its low price	
M3.3	I choose the food I consume, because it is convenient to purchase	
M3.4	I buy fresh vegetables to cook myself more often than frozen	
M3.5	I usually buy food that is easy to prepare	
M3.6	I usually buy food that it is on sale	
M3.7	I prefer to buy food that is ready to eat or pre-cooked	
Social & cultural motivations		
M4.1	Meals are a time of fellowship and pleasure	5-point scale from "Strongly Disagree" (1) to "Strongly Agree" (5)
M4.2	I eat more than usual when I have company	
M4.3	It is important to me that the food I eat is similar to the food I ate when I was a child	
M4.4	I eat certain foods because other people (my colleagues, friends, family) also eat it	
M4.5	I prefer to eat alone	
M4.6	I choose the foods I eat, because it fits the season	
M4.7	I eat certain foods because I am expected to eat them	
M4.8	I like to try new foods to which I am not accustomed	
M4.9	I usually eat food that is trendy	
Environmental and political motivations		
M5.1	It is important to me that the food I eat is prepared/packed in an environmental friendly way	5-point scale from "Strongly Disagree" (1) to "Strongly Agree" (5)
M5.2	When I cook I have in mind the quantities to avoid food waste	
M5.3	It is important to me that the food I eat comes from my own country	
M5.4	I prefer to eat food that has been produced in a way that animals' rights have been respected	
M5.5	I choose foods that have been produced in countries where human rights are not violated	
M5.6	I avoid going to restaurants that do not have a recovery policy of food surplus	
M5.7	I prefer to buy foods that comply with policies of minimal usage of packaging	

Marketing and commercials motivations		
M6.1	When I buy food I usually do not care about the marketing campaigns happening in the shop	5-point scale from "Strongly Disagree" (1) to "Strongly Agree" (5)
M6.2	I eat what I eat, because I recognize it from advertisements or have seen it on TV	
M6.3	I usually buy food that spontaneously appeals to me (e.g. situated at eye level, appealing colors, pleasant packaging)	
M6.4	When I go shopping I prefer to read food labels instead of believing in advertising campaigns	
M6.5	Food advertising campaigns increase my desire to eat certain foods	
M6.6	Brands are important to me when making food choices	
M6.7	I try to schedule my food shopping for when I know there are promotions or discounts	
